# {4-Phenyl-1-[1-(1,3-thia­zol-2-yl)ethyl­idene]­thio­semicarbazidato}{4-phenyl-1-[1-(1,3-thia­zol-2-yl)ethylidene]­thio­semi­carbazide}nickel(II) chloride mono­hydrate

**DOI:** 10.1107/S1600536810013280

**Published:** 2010-04-21

**Authors:** Ramaiyer Venkatraman, Md. Alamgir Hossain, Frank R. Fronczek

**Affiliations:** a Department of Chemistry and Biochemistry, Jackson State University, Jackson, MS 39217, USA; b Department of Chemistry, Louisiana State University, Baton Rouge, LA 70803, USA

## Abstract

In the title compound, [Ni(C_12_H_11_N_4_S_2_)(C_12_H_12_N_4_S_2_)]Cl·H_2_O, the Ni^II^ ion is chelated by two 2-acetyl­thia­zole-3-phenyl­thio­semicarbazone ligands, forming a distorted octa­hedral complex. The metal ion is coordinated *via* the thia­zole nitro­gen, imine nitro­gen and thione sulfur atoms from each thio­semicarbazone ligand, and two coordinating units lie almost perpendicular to each other give dihedral angle = 81.89 (1)°]. One thio­semicarbazone unit is found to bind a chloride anion through two hydrogen bonds, while the other is linked with the disordered crystal water molecule. Two mol­ecules are connected to each other through an inter­molecular N—H⋯S inter­action, forming a centrosymmetric dimer. Dimers are linked into sheets by π–π stacking of two phenyl rings [shortest C⋯C distance = 4.041 (3) Å].

## Related literature

For general background to thio­semicarbazones and their metal complexes, see: Haiduc & Silverstru (1990 ▶); Nath *et al.* (2001 ▶); Padhye & Kauffman (1985 ▶); Pellerito & Nagy (2002 ▶); Ali & Livingstone (1974 ▶); Barros-García *et al.* (2005 ▶); Campbell (1975 ▶). For related structures, see: Ketcham *et al.* (2002 ▶); Lima *et al.* (1999 ▶); Viñuelas-Zahínos *et al.* (2008 ▶); Saeed *et al.* (2009 ▶); Venkatraman *et al.* (2009 ▶).
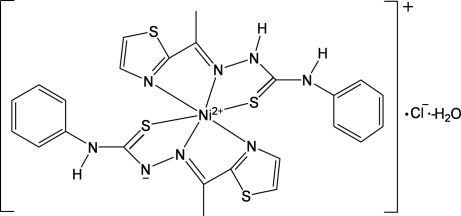


## Experimental

### 

#### Crystal data

[Ni(C_12_H_11_N_4_S_2_)(C_12_H_12_N_4_S_2_)]Cl·H_2_O
*M* *_r_* = 663.92
Triclinic, 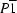

*a* = 8.5983 (15) Å
*b* = 12.929 (2) Å
*c* = 13.492 (2) Å
α = 101.710 (8)°
β = 90.168 (8)°
γ = 98.946 (7)°
*V* = 1449.9 (4) Å^3^
*Z* = 2
Mo *K*α radiation
μ = 1.08 mm^−1^
*T* = 90 K
0.33 × 0.27 × 0.08 mm

#### Data collection

Nonius KappaCCD diffractometer
Absorption correction: multi-scan (*SCALEPACK*; Otwinowski & Minor, 1997 ▶) *T*_min_ = 0.716, *T*_max_ = 0.918
31053 measured reflections
8823 independent reflections
7120 reflections with *I* > 2σ(*I*)
*R*_int_ = 0.027

#### Refinement

*R*[*F* ^2^ > 2σ(*F* ^2^)] = 0.034
*wR*(*F* ^2^) = 0.081
*S* = 1.03
8823 reflections
374 parameters
3 restraints
H atoms treated by a mixture of independent and constrained refinement
Δρ_max_ = 0.46 e Å^−3^
Δρ_min_ = −0.62 e Å^−3^

### 

Data collection: *COLLECT* (Nonius 2000 ▶); cell refinement: *DENZO* and *SCALEPACK* (Otwinowski & Minor, 1997 ▶); data reduction: *DENZO* and *SCALEPACK*; program(s) used to solve structure: *SIR97* (Altomare *et al.*, 1999 ▶); program(s) used to refine structure: *SHELXL97* (Sheldrick, 2008 ▶); molecular graphics: *ORTEP-3 for Windows* (Farrugia, 1997 ▶); software used to prepare material for publication: *SHELXL97*.

## Supplementary Material

Crystal structure: contains datablocks global, I. DOI: 10.1107/S1600536810013280/br2143sup1.cif

Structure factors: contains datablocks I. DOI: 10.1107/S1600536810013280/br2143Isup2.hkl

Additional supplementary materials:  crystallographic information; 3D view; checkCIF report

## Figures and Tables

**Table 1:** Hydrogen-bond geometry (Å, °)

*D*—H⋯*A*	*D*—H	H⋯*A*	*D*⋯*A*	*D*—H⋯*A*
N4—H4*N*⋯S2^i^	0.80 (2)	2.54 (2)	3.2595 (15)	150.7 (19)
N7—H7*N*⋯Cl1	0.82 (2)	2.45 (2)	3.2050 (15)	153.9 (19)
N8—H8*N*⋯Cl1	0.89 (2)	2.23 (2)	3.1051 (16)	168.5 (19)
O1—H01⋯Cl1^ii^	0.84 (2)	2.33 (2)	3.1653 (19)	178 (3)
O1—H02⋯N3	0.82 (2)	2.40 (2)	3.112 (2)	146 (3)

Symmetry codes: (i) 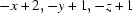; (ii) 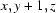.

**Fig. 1.**
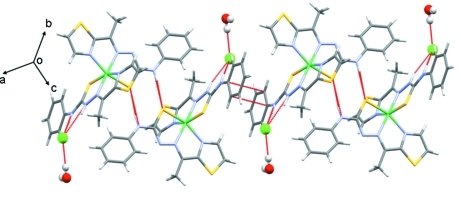
Packing diagram of the title compound showing a molecular chain viewed along a axis.

## supplementary crystallographic information

### Comment

Studies on thiosemicarbazones and their metal complexes remain an active field of research for more than three decades due to their significant impacts in biology and chemistry (Ali & Livingstone, 1974; Campbell, 1975; Haiduc & Silverstru, 1990; Nath *et al.*, 2001; Padhye & Kauffman, 1985; Pellerito & Nagy, 2002). Thiosemicarbazones are also known to stabilize uncommon oxidation states of metals upon complexation. The variation of coordination numbers exhibited by transition metals in these complexes is utilized in various redox reactions and found to inhibit the activity of metalloenzymes. In particular, the characterization of the coordination aspects of metal complexes with thiosemicarbazone ligands are important in order to model the physical and chemical behaviour of metalloenzymes (Viñuelas-Zahínos *et al.* 2008). Nickel(II) complexes of heterocyclic thiosemicarbazones were previously reported by Ketcham *et al.* (2002) and de Lima *et al.* (1999). Recently, Barros-Garcia *et al.* (2005) studied the structural and ligation properties of 2-acetyl thiazole semicarbazone of nickel(II). In the present study, we report the synthesis and structure of nickel(II) complex of the phenyl derivative of 2-acetylthiazole-3-thiosemicarbazone.

The title complex is a result of interaction between the neutral ligand molecules and nickel (II) ions in aqueous solution. In the complex, nickel(II) is chelated by two 2-acetylthiazole-3-phenylthiosemicarbazone ligands forming an octahedral complex (Fig. 1). The central metal ion binds via thiazole nitrogen, imine nitrogen and thione sulfur from each thiosemicarbazone. The coordination of the two ligands is meridonal due to the strong tendency toward planarity by heterocyclic thiosemicarbazones. The nickel ion coordinates with the two imine nitrogen with the Ni···N distances of 2.0332 (13) and 2.0479 (14) Å which are shorter than the corresponding distances of ring nitrogens (2.0967 (14) and 2.1076 (14) Å ). All these four bonds are shorter than the Ni···S distances (2.3619 (5) and 2.4139 (5) Å ). The central metal ion is distorted from octahedral symmetry as indicated by the angles N1—Ni1—S2, 160.44 (4)° and N5—Ni1—S4, 159.14 (4)°.

Indeed, the participation of sulfur groups as electron pair donors in coordinating Ni^II^ ion makes the secondary amines more acidic which results the complete loss of one proton on N3 from one ligand. Interestingly, this nitrogen (N3) acts as a hydrogen bond acceptor for one water molecule (Table 1). On the other hand, the second ligand is involved in binding a chloride anion with two hydrogen bonds (NH···Cl = 3.2050 (15) and 3.1051 (16) Å, which are slightly shorter than 3.048 (3) Å observed in a cryptand based receptor binding a chloride anion in its cavity (Saeed *et al.*, 2009). Additionally, the two neighboring molecules are found to form a centrosymmetric dimer through NH···S interactions (Fig. 2). In the packing diagram the dimers are again connected with π-π stacking of two phenyl rings (Fig. 3).

The structure contains an unreasonably short distance O1A···C17, 2.629 (18) Å. This distance involves an atom (O1A) which was treated as occupied <9%. Since the contact is to the average position of a fully-occupied atom (C17), the distance does not imply an actual contact between two atoms. After refinement of the ordered part of the structure, residual density of 1.08 eÅ^-3^ was located in a cavity slightly too small for occupancy by a water molecule. The site is 2.488 (17) Å from O1 (at 1-x, 1-y, 1-z), and is taken to be an alternate site for O1. Refinement with O1 and O1A having occupancies summing to unity led to occupancy of 0.087 (4) for O1A. The cavity likely expands when O1A is occupied, and the displacement parameters of the atoms surrounding the cavity, including C17, support this interpretation. The environments of water molecule O1 and site O1A are quite different, the former engaging in long hydrogen bonds with N and Cl, while the latter is in a small void with no hydrogen bonding. This accounts for the large difference in the refined occupancies of the two sites. It appears unlikely that both sites could be simultaneously occupied, because of the short distance between them.

### Experimental

The cationic nickel complex was prepared by adding an aqueous solution of nickel (II) chloride to a boiling methanolic solution of thiosemicarbazone (Venkatraman *et al.* 2009) in 1:2 mol ratio. Heating was continued for about 2 hours. Light brown colored crystals were obtained by evaporation of the solvent at room temperature (yield = 60%).

### Refinement

H atoms on C were placed in idealized positions with C—H distances 0.95 - 0.98 Å and thereafter treated as riding. The coordinates of those on N and O were refined. *U*_iso_ for H was assigned as 1.2 times *U*_eq_ of the attached atom (1.5 for methyl). A torsional parameter was refined for each methyl group. A residual peak of density 1.08 eÅ^-3^, with nearest distance 2.5 Å to the water position was interpreted as a disordered water site. The partially-occupied water site O1A was treated as isotropic, and its H atoms were not located. The largest residual density peak was 0.84 Å from S3.

### Crystal data

**Table:**

[Ni(C_12_H_11_N_4_S_2_)(C_12_H_12_N_4_S_2_)]Cl·H_2_O	*Z* = 2
*M**_r_* = 663.92	*F*(000) = 684
Triclinic, *P*1	*D*_x_ = 1.521 Mg m^−^^3^
Hall symbol: -P 1	Mo *K*α radiation, λ = 0.71073 Å
*a* = 8.5983 (15) Å	Cell parameters from 8157 reflections
*b* = 12.929 (2) Å	θ = 2.5–30.5°
*c* = 13.492 (2) Å	µ = 1.08 mm^−^^1^
α = 101.710 (8)°	*T* = 90 K
β = 90.168 (8)°	Fragment, dark orange-red
γ = 98.946 (7)°	0.33 × 0.27 × 0.08 mm
*V* = 1449.9 (4) Å^3^	

### Data collection

**Table:**

Nonius KappaCCD diffractometer with an Oxford Cryosystems Cryostream cooler	8823 independent reflections
Radiation source: fine-focus sealed tube	7120 reflections with *I* > 2σ(*I*)
graphite	*R*_int_ = 0.027
ω and φ scans	θ_max_ = 30.5°, θ_min_ = 2.6°
Absorption correction: multi-scan (*SCALEPACK*; Otwinowski & Minor, 1997)	*h* = −11→12
*T*_min_ = 0.716, *T*_max_ = 0.918	*k* = −18→18
31053 measured reflections	*l* = −18→19

### Refinement

**Table:**

Refinement on *F*^2^	Primary atom site location: structure-invariant direct methods
Least-squares matrix: full	Secondary atom site location: difference Fourier map
*R*[*F*^2^ > 2σ(*F*^2^)] = 0.034	Hydrogen site location: inferred from neighbouring sites
*wR*(*F*^2^) = 0.081	H atoms treated by a mixture of independent and constrained refinement
*S* = 1.03	*w* = 1/[σ^2^(*F*_o_^2^) + (0.0317*P*)^2^ + 0.7508*P*] where *P* = (*F*_o_^2^ + 2*F*_c_^2^)/3
8823 reflections	(Δ/σ)_max_ = 0.001
374 parameters	Δρ_max_ = 0.46 e Å^−^^3^
3 restraints	Δρ_min_ = −0.62 e Å^−^^3^

### Special details

**Table:**

Geometry. All esds (except the esd in the dihedral angle between two l.s. planes) are estimated using the full covariance matrix. The cell esds are taken into account individually in the estimation of esds in distances, angles and torsion angles; correlations between esds in cell parameters are only used when they are defined by crystal symmetry. An approximate (isotropic) treatment of cell esds is used for estimating esds involving l.s. planes.
Refinement. Refinement of *F*^2^ against ALL reflections. The weighted *R*-factor wR and goodness of fit *S* are based on *F*^2^, conventional *R*-factors *R* are based on *F*, with *F* set to zero for negative *F*^2^. The threshold expression of *F*^2^ > σ(*F*^2^) is used only for calculating *R*-factors(gt) etc. and is not relevant to the choice of reflections for refinement. *R*-factors based on *F*^2^ are statistically about twice as large as those based on *F*, and *R*- factors based on ALL data will be even larger.

### Fractional atomic coordinates and isotropic or equivalent isotropic displacement parameters (Å^2^)

**Table:**

	*x*	*y*	*z*	*U*_iso_*/*U*_eq_	Occ. (<1)
Ni1	0.73755 (2)	0.394293 (16)	0.219421 (15)	0.01278 (5)	
S1	0.69340 (6)	0.47956 (4)	−0.08422 (3)	0.02390 (10)	
S2	0.85983 (5)	0.45716 (3)	0.38207 (3)	0.01606 (8)	
S3	0.28921 (6)	0.38933 (4)	0.38582 (5)	0.03629 (13)	
S4	0.93712 (5)	0.28513 (3)	0.16410 (3)	0.01845 (9)	
N1	0.66738 (17)	0.38969 (11)	0.06858 (10)	0.0168 (3)	
N2	0.84148 (16)	0.54131 (10)	0.20123 (10)	0.0145 (3)	
N3	0.92388 (16)	0.61821 (11)	0.27600 (10)	0.0160 (3)	
N4	1.01213 (17)	0.65179 (11)	0.44242 (10)	0.0168 (3)	
H4N	1.009 (2)	0.6270 (17)	0.4923 (16)	0.020*	
N5	0.52775 (16)	0.43701 (11)	0.28172 (10)	0.0160 (3)	
N6	0.61975 (16)	0.25083 (11)	0.24132 (10)	0.0155 (3)	
N7	0.68555 (17)	0.16005 (11)	0.21622 (11)	0.0189 (3)	
H7N	0.641 (2)	0.1023 (18)	0.2257 (15)	0.023*	
N8	0.89284 (18)	0.07572 (12)	0.17390 (12)	0.0204 (3)	
H8N	0.823 (3)	0.0229 (18)	0.1878 (16)	0.024*	
C1	0.5889 (2)	0.31759 (14)	−0.01143 (13)	0.0208 (3)	
H1	0.5375	0.2487	−0.0053	0.025*	
C2	0.5902 (2)	0.35226 (14)	−0.10032 (13)	0.0238 (4)	
H2	0.5411	0.3118	−0.1623	0.029*	
C3	0.7296 (2)	0.47931 (13)	0.04134 (12)	0.0175 (3)	
C4	0.8216 (2)	0.56675 (13)	0.11410 (12)	0.0178 (3)	
C5	0.8832 (3)	0.67237 (14)	0.08888 (13)	0.0272 (4)	
H5A	0.8206	0.7259	0.1221	0.041*	
H5B	0.8759	0.6663	0.0154	0.041*	
H5C	0.9935	0.6943	0.1126	0.041*	
C6	0.93577 (18)	0.58278 (12)	0.36129 (12)	0.0142 (3)	
C7	1.0919 (2)	0.75756 (13)	0.45124 (12)	0.0184 (3)	
C8	1.1888 (3)	0.78887 (16)	0.37662 (15)	0.0314 (4)	
H8	1.1962	0.7405	0.3142	0.038*	
C9	1.2748 (3)	0.89174 (17)	0.39429 (16)	0.0383 (5)	
H9	1.3432	0.9129	0.3443	0.046*	
C10	1.2617 (3)	0.96389 (17)	0.48438 (16)	0.0390 (5)	
H10	1.3183	1.0347	0.4952	0.047*	
C11	1.1656 (3)	0.93179 (18)	0.55803 (16)	0.0430 (6)	
H11	1.1561	0.9806	0.6198	0.052*	
C12	1.0830 (3)	0.82842 (16)	0.54207 (14)	0.0334 (5)	
H12	1.0199	0.8061	0.5939	0.040*	
C13	0.4732 (2)	0.53104 (14)	0.32056 (13)	0.0203 (3)	
H13	0.5221	0.5984	0.3088	0.024*	
C14	0.3436 (2)	0.51963 (16)	0.37710 (15)	0.0288 (4)	
H14	0.2905	0.5765	0.4077	0.035*	
C15	0.44159 (19)	0.35541 (14)	0.31114 (13)	0.0188 (3)	
C16	0.4887 (2)	0.24947 (13)	0.28841 (13)	0.0195 (3)	
C17	0.3995 (2)	0.15581 (15)	0.32400 (18)	0.0336 (5)	
H17A	0.3441	0.1050	0.2661	0.050*	
H17B	0.3228	0.1804	0.3733	0.050*	
H17C	0.4731	0.1206	0.3559	0.050*	
C18	0.83731 (19)	0.16880 (13)	0.18405 (12)	0.0167 (3)	
C19	1.0377 (2)	0.04743 (14)	0.13910 (13)	0.0198 (3)	
C20	1.1345 (2)	0.09939 (15)	0.07580 (14)	0.0236 (4)	
H20	1.1079	0.1613	0.0564	0.028*	
C21	1.2697 (2)	0.06037 (15)	0.04129 (15)	0.0265 (4)	
H21	1.3359	0.0961	−0.0017	0.032*	
C22	1.3101 (2)	−0.03042 (15)	0.06854 (15)	0.0274 (4)	
H22	1.4035	−0.0564	0.0447	0.033*	
C23	1.2126 (2)	−0.08261 (15)	0.13097 (15)	0.0263 (4)	
H23	1.2389	−0.1450	0.1495	0.032*	
C24	1.0774 (2)	−0.04432 (14)	0.16631 (14)	0.0226 (4)	
H24	1.0113	−0.0803	0.2091	0.027*	
Cl1	0.61127 (5)	−0.09146 (3)	0.21252 (3)	0.02521 (9)	
O1	0.6845 (2)	0.76617 (14)	0.36670 (17)	0.0455 (6)	0.913 (4)
H01	0.662 (3)	0.804 (2)	0.326 (2)	0.068*	
H02	0.768 (3)	0.754 (2)	0.342 (2)	0.068*	
O1A	0.524 (2)	0.2403 (15)	0.5062 (14)	0.041 (6)*	0.087 (4)

### Atomic displacement parameters (Å^2^)

**Table:**

	*U*^11^	*U*^22^	*U*^33^	*U*^12^	*U*^13^	*U*^23^
Ni1	0.01436 (10)	0.01070 (9)	0.01427 (10)	0.00209 (7)	0.00101 (7)	0.00476 (7)
S1	0.0373 (3)	0.0193 (2)	0.01466 (19)	0.00106 (18)	−0.00456 (17)	0.00520 (15)
S2	0.01935 (19)	0.01415 (18)	0.01562 (18)	0.00036 (14)	−0.00207 (14)	0.00703 (14)
S3	0.0315 (3)	0.0256 (2)	0.0568 (3)	0.0120 (2)	0.0271 (2)	0.0142 (2)
S4	0.01769 (19)	0.01322 (18)	0.0258 (2)	0.00322 (14)	0.00702 (16)	0.00654 (15)
N1	0.0201 (7)	0.0132 (6)	0.0174 (6)	0.0027 (5)	−0.0008 (5)	0.0038 (5)
N2	0.0172 (6)	0.0116 (6)	0.0148 (6)	0.0013 (5)	0.0011 (5)	0.0036 (5)
N3	0.0205 (7)	0.0120 (6)	0.0152 (6)	−0.0004 (5)	−0.0020 (5)	0.0041 (5)
N4	0.0221 (7)	0.0147 (6)	0.0137 (6)	−0.0005 (5)	−0.0008 (5)	0.0057 (5)
N5	0.0164 (6)	0.0161 (6)	0.0164 (6)	0.0045 (5)	−0.0003 (5)	0.0037 (5)
N6	0.0161 (6)	0.0122 (6)	0.0185 (6)	0.0030 (5)	0.0004 (5)	0.0034 (5)
N7	0.0192 (7)	0.0099 (6)	0.0288 (8)	0.0025 (5)	0.0060 (6)	0.0066 (6)
N8	0.0206 (7)	0.0138 (7)	0.0296 (8)	0.0055 (5)	0.0081 (6)	0.0089 (6)
C1	0.0238 (8)	0.0153 (8)	0.0220 (8)	0.0027 (6)	−0.0033 (7)	0.0013 (6)
C2	0.0317 (10)	0.0181 (8)	0.0196 (8)	0.0028 (7)	−0.0055 (7)	0.0004 (6)
C3	0.0230 (8)	0.0164 (8)	0.0141 (7)	0.0035 (6)	−0.0002 (6)	0.0052 (6)
C4	0.0238 (8)	0.0146 (7)	0.0157 (7)	0.0006 (6)	0.0000 (6)	0.0069 (6)
C5	0.0435 (11)	0.0185 (8)	0.0185 (8)	−0.0065 (8)	−0.0041 (8)	0.0098 (7)
C6	0.0140 (7)	0.0135 (7)	0.0163 (7)	0.0035 (6)	0.0009 (6)	0.0045 (6)
C7	0.0214 (8)	0.0161 (8)	0.0170 (7)	−0.0011 (6)	−0.0034 (6)	0.0049 (6)
C8	0.0393 (11)	0.0248 (10)	0.0256 (9)	−0.0047 (8)	0.0090 (8)	0.0024 (8)
C9	0.0487 (13)	0.0307 (11)	0.0298 (10)	−0.0144 (10)	0.0076 (9)	0.0085 (8)
C10	0.0541 (14)	0.0257 (10)	0.0294 (10)	−0.0187 (10)	−0.0043 (10)	0.0067 (8)
C11	0.0641 (16)	0.0277 (11)	0.0253 (10)	−0.0162 (10)	0.0042 (10)	−0.0047 (8)
C12	0.0462 (12)	0.0261 (10)	0.0209 (9)	−0.0108 (9)	0.0075 (8)	0.0013 (7)
C13	0.0239 (8)	0.0179 (8)	0.0212 (8)	0.0076 (7)	0.0003 (7)	0.0056 (6)
C14	0.0314 (10)	0.0230 (9)	0.0362 (10)	0.0145 (8)	0.0110 (8)	0.0083 (8)
C15	0.0155 (7)	0.0190 (8)	0.0227 (8)	0.0042 (6)	0.0041 (6)	0.0052 (6)
C16	0.0164 (8)	0.0166 (8)	0.0262 (8)	0.0020 (6)	0.0045 (6)	0.0066 (6)
C17	0.0293 (10)	0.0198 (9)	0.0539 (13)	0.0035 (8)	0.0218 (9)	0.0127 (9)
C18	0.0183 (8)	0.0149 (7)	0.0176 (7)	0.0033 (6)	0.0026 (6)	0.0045 (6)
C19	0.0184 (8)	0.0161 (8)	0.0256 (8)	0.0051 (6)	0.0042 (6)	0.0043 (6)
C20	0.0269 (9)	0.0202 (8)	0.0267 (9)	0.0087 (7)	0.0084 (7)	0.0082 (7)
C21	0.0252 (9)	0.0227 (9)	0.0339 (10)	0.0067 (7)	0.0112 (8)	0.0088 (8)
C22	0.0223 (9)	0.0245 (9)	0.0370 (10)	0.0095 (7)	0.0090 (8)	0.0054 (8)
C23	0.0228 (9)	0.0195 (8)	0.0395 (11)	0.0076 (7)	0.0047 (8)	0.0099 (8)
C24	0.0227 (8)	0.0155 (8)	0.0316 (9)	0.0039 (6)	0.0059 (7)	0.0086 (7)
Cl1	0.0259 (2)	0.0181 (2)	0.0311 (2)	−0.00051 (16)	0.00502 (17)	0.00675 (16)
O1	0.0389 (11)	0.0279 (9)	0.0777 (15)	0.0110 (8)	0.0042 (10)	0.0253 (9)

### Geometric parameters (Å, °)

**Table:**

Ni1—N2	2.0332 (13)	C5—H5B	0.9800
Ni1—N6	2.0479 (14)	C5—H5C	0.9800
Ni1—N5	2.0967 (14)	C7—C12	1.383 (3)
Ni1—N1	2.1076 (14)	C7—C8	1.392 (3)
Ni1—S2	2.3619 (5)	C8—C9	1.392 (3)
Ni1—S4	2.4139 (5)	C8—H8	0.9500
S1—C2	1.7155 (19)	C9—C10	1.391 (3)
S1—C3	1.7223 (16)	C9—H9	0.9500
S2—C6	1.7317 (16)	C10—C11	1.381 (3)
S3—C14	1.705 (2)	C10—H10	0.9500
S3—C15	1.7121 (17)	C11—C12	1.387 (3)
S4—C18	1.6837 (17)	C11—H11	0.9500
N1—C3	1.321 (2)	C12—H12	0.9500
N1—C1	1.372 (2)	C13—C14	1.358 (3)
N2—C4	1.301 (2)	C13—H13	0.9500
N2—N3	1.3703 (18)	C14—H14	0.9500
N3—C6	1.331 (2)	C15—C16	1.462 (2)
N4—C6	1.357 (2)	C16—C17	1.495 (2)
N4—C7	1.413 (2)	C17—H17A	0.9800
N4—H4N	0.80 (2)	C17—H17B	0.9800
N5—C15	1.322 (2)	C17—H17C	0.9800
N5—C13	1.376 (2)	C19—C20	1.391 (2)
N6—C16	1.294 (2)	C19—C24	1.400 (2)
N6—N7	1.3633 (19)	C20—C21	1.383 (2)
N7—C18	1.372 (2)	C20—H20	0.9500
N7—H7N	0.82 (2)	C21—C22	1.391 (3)
N8—C18	1.344 (2)	C21—H21	0.9500
N8—C19	1.408 (2)	C22—C23	1.388 (3)
N8—H8N	0.89 (2)	C22—H22	0.9500
C1—C2	1.362 (2)	C23—C24	1.382 (2)
C1—H1	0.9500	C23—H23	0.9500
C2—H2	0.9500	C24—H24	0.9500
C3—C4	1.461 (2)	O1—H01	0.837 (17)
C4—C5	1.492 (2)	O1—H02	0.819 (17)
C5—H5A	0.9800		
			
N2—Ni1—N6	176.08 (5)	N3—C6—S2	126.98 (12)
N2—Ni1—N5	98.26 (5)	N4—C6—S2	115.26 (11)
N6—Ni1—N5	77.83 (5)	C12—C7—C8	119.82 (17)
N2—Ni1—N1	78.96 (5)	C12—C7—N4	117.41 (15)
N6—Ni1—N1	101.11 (5)	C8—C7—N4	122.49 (16)
N5—Ni1—N1	95.07 (5)	C7—C8—C9	119.35 (18)
N2—Ni1—S2	81.48 (4)	C7—C8—H8	120.3
N6—Ni1—S2	98.44 (4)	C9—C8—H8	120.3
N5—Ni1—S2	88.16 (4)	C10—C9—C8	120.67 (19)
N1—Ni1—S2	160.44 (4)	C10—C9—H9	119.7
N2—Ni1—S4	102.43 (4)	C8—C9—H9	119.7
N6—Ni1—S4	81.48 (4)	C11—C10—C9	119.44 (19)
N5—Ni1—S4	159.14 (4)	C11—C10—H10	120.3
N1—Ni1—S4	91.49 (4)	C9—C10—H10	120.3
S2—Ni1—S4	92.252 (19)	C10—C11—C12	120.2 (2)
C2—S1—C3	89.79 (8)	C10—C11—H11	119.9
C6—S2—Ni1	95.09 (5)	C12—C11—H11	119.9
C14—S3—C15	89.67 (9)	C7—C12—C11	120.49 (18)
C18—S4—Ni1	97.18 (6)	C7—C12—H12	119.8
C3—N1—C1	111.36 (14)	C11—C12—H12	119.8
C3—N1—Ni1	109.88 (11)	C14—C13—N5	114.44 (16)
C1—N1—Ni1	138.41 (12)	C14—C13—H13	122.8
C4—N2—N3	117.28 (13)	N5—C13—H13	122.8
C4—N2—Ni1	117.92 (11)	C13—C14—S3	110.81 (14)
N3—N2—Ni1	124.70 (10)	C13—C14—H14	124.6
C6—N3—N2	111.60 (13)	S3—C14—H14	124.6
C6—N4—C7	130.21 (14)	N5—C15—C16	120.30 (15)
C6—N4—H4N	112.7 (15)	N5—C15—S3	114.19 (13)
C7—N4—H4N	117.1 (15)	C16—C15—S3	125.19 (13)
C15—N5—C13	110.87 (15)	N6—C16—C15	111.47 (15)
C15—N5—Ni1	111.02 (11)	N6—C16—C17	125.99 (16)
C13—N5—Ni1	136.07 (12)	C15—C16—C17	122.37 (15)
C16—N6—N7	120.21 (14)	C16—C17—H17A	109.5
C16—N6—Ni1	118.79 (11)	C16—C17—H17B	109.5
N7—N6—Ni1	120.66 (11)	H17A—C17—H17B	109.5
N6—N7—C18	118.32 (14)	C16—C17—H17C	109.5
N6—N7—H7N	121.7 (15)	H17A—C17—H17C	109.5
C18—N7—H7N	119.6 (15)	H17B—C17—H17C	109.5
C18—N8—C19	130.77 (15)	N8—C18—N7	111.65 (14)
C18—N8—H8N	113.2 (14)	N8—C18—S4	126.23 (13)
C19—N8—H8N	115.9 (14)	N7—C18—S4	122.10 (12)
C2—C1—N1	114.90 (16)	C20—C19—C24	119.69 (16)
C2—C1—H1	122.5	C20—C19—N8	124.65 (15)
N1—C1—H1	122.5	C24—C19—N8	115.51 (15)
C1—C2—S1	110.19 (13)	C21—C20—C19	119.57 (16)
C1—C2—H2	124.9	C21—C20—H20	120.2
S1—C2—H2	124.9	C19—C20—H20	120.2
N1—C3—C4	120.81 (14)	C20—C21—C22	120.99 (17)
N1—C3—S1	113.76 (12)	C20—C21—H21	119.5
C4—C3—S1	125.42 (12)	C22—C21—H21	119.5
N2—C4—C3	112.28 (14)	C23—C22—C21	119.28 (17)
N2—C4—C5	125.17 (15)	C23—C22—H22	120.4
C3—C4—C5	122.55 (14)	C21—C22—H22	120.4
C4—C5—H5A	109.5	C24—C23—C22	120.36 (17)
C4—C5—H5B	109.5	C24—C23—H23	119.8
H5A—C5—H5B	109.5	C22—C23—H23	119.8
C4—C5—H5C	109.5	C23—C24—C19	120.11 (17)
H5A—C5—H5C	109.5	C23—C24—H24	119.9
H5B—C5—H5C	109.5	C19—C24—H24	119.9
N3—C6—N4	117.75 (14)	H01—O1—H02	96 (2)
			
N6—Ni1—N1—C1	−9.68 (18)	C6—N4—C7—C8	−41.3 (3)
N2—Ni1—N5—C13	−12.01 (16)	Ni1—N5—C15—C16	−6.61 (19)
Ni1—N1—C3—C4	−4.3 (2)	Ni1—N6—C16—C15	4.66 (19)
Ni1—N2—C4—C3	−2.43 (19)	Ni1—N6—N7—C18	5.4 (2)
Ni1—N2—N3—C6	3.64 (18)	N5—C15—C16—N6	1.6 (2)
N1—C3—C4—N2	4.6 (2)	C19—N8—C18—S4	−4.7 (3)
N2—N3—C6—S2	−0.3 (2)	N6—N7—C18—S4	−6.0 (2)
C7—N4—C6—N3	−3.9 (3)	Ni1—S4—C18—N7	3.48 (14)
Ni1—S2—C6—N3	−2.24 (15)	C18—N8—C19—C20	−23.1 (3)

### Hydrogen-bond geometry (Å, °)

**Table:**

*D*—H···*A*	*D*—H	H···*A*	*D*···*A*	*D*—H···*A*
N4—H4N···S2^i^	0.80 (2)	2.54 (2)	3.2595 (15)	150.7 (19)
N7—H7N···Cl1	0.82 (2)	2.45 (2)	3.2050 (15)	153.9 (19)
N8—H8N···Cl1	0.89 (2)	2.23 (2)	3.1051 (16)	168.5 (19)
O1—H01···Cl1^ii^	0.84 (2)	2.33 (2)	3.1653 (19)	178 (3)
O1—H02···N3	0.82 (2)	2.40 (2)	3.112 (2)	146 (3)

Symmetry codes: (i) −*x*+2, −*y*+1, −*z*+1; (ii) *x*, *y*+1, *z*.
